# Supplementary material for: The interactome of intact mitochondria by cross-linking mass spectrometry provides evidence for coexisting respiratory supercomplexes
Source: Mol Cell Proteomics. 2017 Dec 8;17(2):216–32. doi: 10.1074/mcp.RA117.000470 (PMC5795388; doi:10.1074/mcp.RA117.000470)
Supplement: Supplemental Data [file supp_RA117.000470_133922_0_supp_23978_fzffwf.zip › spectra_annotation/mito_DR_spectra_annotation/217-1-2-1-13-1.pdf]

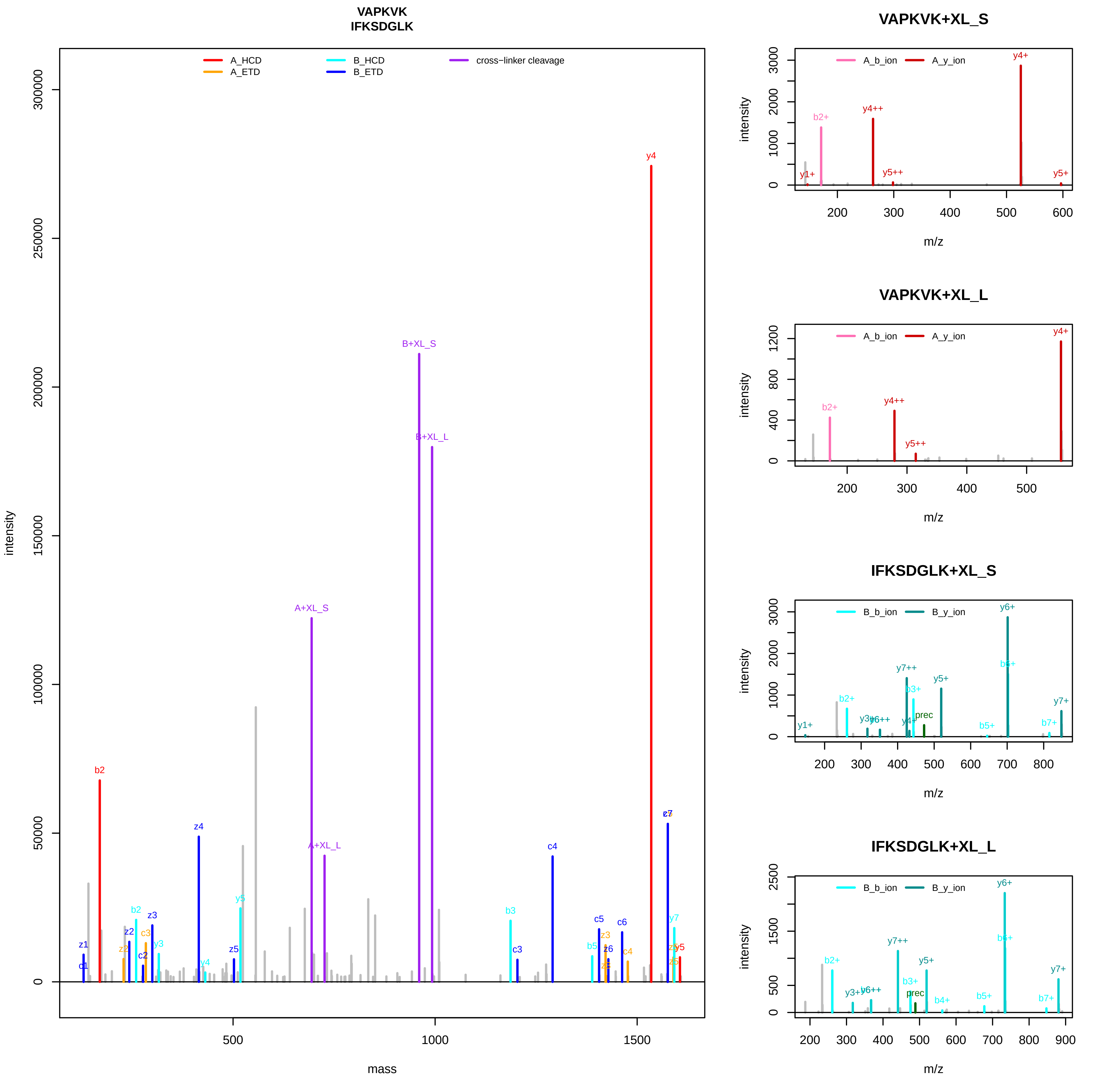
VAPKVK
IFKSDGLK
A_HCD
A_ETD
B_HCD
B_ETD
cross−linker cleavage
intensity
mass
B+XL_S
B+XL_L
A+XL_S
A+XL_L
y4
b2
z4
c4
c5
c6
b3
c3
y5
z7
y7
VAPKVK+XL_S
A_b_ion
A_y_ion
y4+
y4++
b2+
y5++
y1+
y5+
m/z
VAPKVK+XL_L
IFKSDGLK+XL_S
B_b_ion
B_y_ion
y6+
y7++
b6+
y5+
b3+
b2+
y7+
prec
b5+
b7+
y1+
IFKSDGLK+XL_L
